# Extensive Variation in Cadmium Tolerance and Accumulation among Populations of *Chamaecrista fasciculata*

**DOI:** 10.1371/journal.pone.0063200

**Published:** 2013-05-07

**Authors:** Tessa M. Henson, Wendy Cory, Matthew T. Rutter

**Affiliations:** 1 Department of Biology, College of Charleston, Charleston, South Carolina, United States of America; 2 Department of Chemistry and Biochemistry, College of Charleston, Charleston, South Carolina, United States of America; Instituto Nacional de Cardiologia, Mexico

## Abstract

Plant populations may vary substantially in their tolerance for and accumulation of heavy metals, and assessment of this variability is important when selecting species to use in restoration or phytoremediation projects. We examined the population variation in cadmium tolerance and accumulation in a leguminous pioneer species native to the eastern United States, the partridge pea (*Chamaecrista fasciculata*). We assayed growth, reproduction and patterns of cadmium accumulation in six populations of *C. fasciculata* grown on a range of cadmium-contaminated soils. In general, *C. fasciculata* exhibited tolerance in low to moderate soil cadmium concentrations. Both tolerance and accumulation patterns varied across populations. *C. fasciculata* exhibited many characteristics of a hyperaccumulator species, with high cadmium uptake in shoots and roots. However, cadmium was excluded from extrafloral nectar. As a legume with tolerance for moderate cadmium contamination, *C. fasciculata* has potential for phytoremediation. However, our findings also indicate the importance of considering the effects of genetic variation on plant performance when screening plant populations for utilization in remediation and restoration activities. Also, there is potential for cadmium contamination to affect other species through contamination of leaves, fruits, flowers, pollen and root nodules.

## Introduction

Cadmium (Cd) is a heavy metal that occurs naturally in the environment in concentrations generally <1 mg/kg [1]. It is utilized in industrial processes such as metal plating and manufacture of nickel-cadmium batteries, is a by-product of zinc mining and production, and is a component of phosphate fertilizers and pesticides [2]. Significant amounts of cadmium have been released into the environment as agricultural runoff, industrial waste and household waste, with soil contamination levels ranging from 1 mg/kg to levels in excess of 1020 mg/kg [3]. Unlike other heavy metals such as zinc, copper and nickel, cadmium is a nonessential element in plant nutrition and is highly toxic to both plants and animals at much lower concentrations than other heavy metals and is a human carcinogen [1], [4], [5]. If present in soil or substrate, cadmium is readily taken up by the roots and can be distributed throughout the plant, and can depress uptake of other essential plant nutrients [1], [6]. Plant response to elevated soil cadmium levels includes reduced plant height, biomass, leaf number, flower or fruit number, or death at relatively low concentrations [7].

Because of the problems caused by toxicity of cadmium at contaminated sites, there is considerable interest in phytoremediation strategies (phytoextraction or phytostabilization) that utilize green plants to sequester, uptake, or degrade cadmium compounds. Selection of plants for phytoremediation requires assessment of tolerance and accumulating ability, biogeographic distribution, and availability of propagules [8], [9]. Tolerance is defined as the degree to which plants can withstand exposure to elevated soil cadmium levels without exhibiting phytotoxicity [10]. Tolerance can be evaluated through monitoring the decrease in plant growth or fitness as the level of soil contamination increases. However, tolerance is not a character that can be unambiguously scored by comparison with an objective standard [11]. Plant species vary considerably in cadmium tolerance [2], [7], [12], with a phytoxicity range anywhere between 2.5 to >640 ppm [1], [6], [13].

Partitioning of cadmium throughout the plant is also highly variable across species [7], [14]–[16]. For example, in *Filipendula ulmaria*, root cadmium concentration exceeded that of the soil, and cadmium concentrations in plant parts decreased in the following order: new roots, old roots, rhizomes, stem leaf-stalks, stems and stem leaf blades, and lastly reproductive organs [14]. In hybrid poplar (*Populus deltoids* x *Populus nigra*), cadmium concentration was highest in plant shoots, followed by roots and then leaves [7]. A study of *Helianthus annuus* found that it accumulated the highest concentration of cadmium in its inflorescences, followed by shoots, then roots [16].

In addition to differences between species, many studies have documented population or genetic variation in heavy metal tolerance or accumulation [17]–[24]. An examination of genetic variation, within and between populations, for zinc accumulation in *Arabidopsis halleri* found that genetic variation for accumulation varied at differing exposure levels, with genetic variation decreasing as zinc soil levels increased [21]. Out of four populations of *Thlaspi caerulescens*, one specific population exhibited lower levels of accumulation of zinc, cadmium and nickel, while other populations demonstrated a 10-60-fold increase in cadmium foliar metal concentrations [25]. Similar results have been found in *Triticum aestivum* L. [26].

In general, few studies have examined heavy metal accumulation in fruit, pollen, and nectar. However, these plant parts are often critical to interspecific interactions and can be a vehicle for trophic transfer. *Vigna unguiculata* accumulated cadmium throughout shoots but excluded cadmium from fruits [15]. Exposure of pollen to low levels of cadmium results in reduced pollen germination and pollen tube growth [27]. Heavy metal concentrations (both essential and nonessential) have been observed in a nectar derivative, honey [28]–[31]. However, to the best of our knowledge, no known studies have assessed effects of heavy metal soil exposure on nectar production or cadmium accumulation in nectar itself.

Legumes can be particularly important in restoration or phytoremediation because they facilitate establishment of other species by naturally increasing nitrogen content in the soil. Many legumes experience growth inhibition in the presence of cadmium. Growth inhibition at leaf or shoot cadmium soil levels above 5–10 mg/kg has been reported in, *Phaseolus vulgaris* and *Bituminaria bituminosa* [23], [32]. However, another legume, *Anthyllus vulneraria* subspecies *carpatica*, is tolerant of cadmium up to 16 mg/kg [33]. Cadmium accumulation in the root nodules of legumes has also received little attention, although inoculation with a metal-resistant rhizobial strain resulted in an increase in cadmium accumulation in *Mimosa pudica* [34].

Here we describe the effects of cadmium contamination on germination, growth, and reproductive characters in the partridge pea, *Chamaecrista fasciculata*– a legume native to the eastern United States that is frequently employed during ecological restoration [35], [36]. *C. fasciculata* grows well in disturbed areas, such as along roadsides [36]. C. fasciculata produces extrafloral nectar, is often consumed by herbivores, and forms nodules in association with rhizobium bacteria [36], [37]. *C. fasciculata* has been previously utilized in restoration activities to revegetate surface mined lands and critically eroding areas [35], [36]. Genetically based differences in both appearance and function have been described for different ecotypes of the species [38]–[40] and within ecotypes [37], [39]. These genetically based differences have contributed to the emergence of *C. fasciculata* as a model legume system outside of the subfamily Papilionoideae [41].

Our objective was to determine whether *C. fasciculata* would serve as a potential plant for ecological restoration of cadmium contaminated soils, to identify populations that were particularly tolerant of cadmium, and to evaluate whether within-plant accumulation patterns could be detrimental to other species that interact with the plant. We had the following research questions. 1) What levels of soil cadmium would inhibit growth and germination in C. fasciculata? 2) Is there population-level variation in tolerance to cadmium? 3) What are the patterns and extent of accumulation within the plant? 4) Do accumulation patterns vary between populations or explain patterns of tolerance? We hypothesized that *C. fasciculata* would be tolerant of levels of cadmium contamination common in roadside environments, but that levels of tolerance would vary across populations. We further hypothesized that *C. fasciculata* would accumulate cadmium, with variable accumulation levels between plant organs and between populations. We hypothesized that C. fasciculata would hyperaccumulate cadmium, with hyperaccumulation being defined as a plant that accumulates more than 100 mg/kg of cadmium [22].

## Materials and Methods

### Plant Material

Seeds from six populations of *Chamaecrista fasciculata* were obtained from the USDA Natural Resource Conservation Service and restoration nurseries throughout the eastern United States: Pennsylvania (PA) and Florida (FL) ecotypes from Ernst Conservation Seed, a Minnesota (MN) population from Prairie Moon Nursery, Comanche (TX) and Riley (KS) ecotypes from USDA NCRS, and a Kentucky (KY) population from RoundStone Seed (Figure S1). These ecotypes were selected in order to capture a wide range of possible geographic differences in plant performance and response. All ecotypes have been developed or selected for use in ecosystem restoration; Comanche (TX) was selected by the USDA as a warm-season legume cover crop for revegetating critical areas and mined lands and was collected from a native population in Throckmorton County, Texas [36]. Riley (KS) was collected from a roadside population in Ashland Bottoms, Kansas and is developed for wildlife habitat improvement, erosion control, and conservation use in several southern states [36]. and the other four populations (PA, FL, MN, KY) are available for purchase through nurseries that specialize in providing seeds for restoration projects.

### Experimental Design

Two experiments were performed to determine the tolerance and accumulating ability of *Chamaecrista fasciculata* in response to elevated soil cadmium levels. Both experiments used seed from three populations (TX, MN, and PA). First, we conducted germination trials to test for cadmium tolerance and between-population variation in tolerance. To initiate the experiment, dry sand (Quikrete model 1113) was weighed and moistened with tap water, and then a CdCl_2_ aqueous solution prepared with deionized water [42] was added to the sand to implement the nine treatment levels: 1, 5, 10, 15, 20, 25, 30, 40, and 50 mg/kg cadmium. The tenth treatment was a control and included sand moistened with tap water. Treatment levels were selected based on results of a pilot experiment that indicated germination of *C. fasciculata* can occur in soil cadmium concentrations of 1, 10, and 30 mg/kg, with germination beginning to sharply decline at 30 mg/kg. The sand was placed in 1.5-inch diameter Hummert TLC Polyform 72-cavity germination trays and arranged in a complete randomized block design, with three blocks and every treatment level and population present in each block.

Seeds from each of the three populations were hand scarified before planting, with one seed per cavity sown into moistened sand. We utilized twelve replicates per population per treatment for a total of 36 seeds per treatment and 360 seeds total. Tap water was utilized as necessary to maintain appropriate soil moisture. We monitored germination success for forty-five days by recording the number of days until germination occurred, as well as the date of germination.

After the germination trials were completed and data were analyzed to determine plant germination success across treatment levels, a second experiment designed to assess tolerance and accumulating ability of cadmium throughout the life cycle of the plant was implemented. A mixture of sand and CdCl_2_ was prepared as above to implement three treatment levels based upon the results of the germination trial: 5 mg/kg, 10 mg/kg, and 15 mg/kg. We had found that there was almost no plant growth at soil levels of cadmium above 20 mg/kg and thus chose soil cadmium concentrations where we could test for tolerance and accumulation. The fourth treatment was a control (C) comprised of sand moistened with tap water.

The sand was placed in 4-inch, 39 in^3^ plastic pots. We hand scarified seeds from each of the six populations and mixed them with the correct species of rhizobium inoculant, which was obtained with the seeds from the restoration nurseries and USDA NCRS, before planting. Two seeds per pot were sown into moistened sand, with twenty pots per population per treatment. Pots were arranged as a complete randomized block design, with four blocks and five pots per population in each block at each treatment level.

Approximately two weeks after first germination, we counted the number of seedlings per pot, and then we transplanted extra seedlings to pots within the same population and treatment where no germination occurred to achieve one plant per pot. Remaining seedlings were thinned to one randomly selected plant per pot for a total of 120 plants per treatment and 480 total plants. After transplantation was completed, we utilized tap water as necessary to maintain appropriate soil moisture, and applied 40 ml of Jack’s Classic^©^ Water Soluble Plant Food to all plants once they reached the two-leaf stage, beginning five days after transplantation concluded. The nutrient solution was applied again to all plants sixty days after transplantation. Green Light© Spinosad organic pesticide was applied once weekly for three weeks to control insect populations, with no signs of phytotoxicity. Plants were staked as necessary.

Beginning approximately two weeks after transplantation, leaf number was recorded weekly. Hand pollinations were conducted to determine whether plants grown in cadmium-contaminated soil were able to produce fruit. Once the plants began to flower, we collected pollen by toothpick from multiple flowering plants, combined it together, and utilized it to pollinate flowers within the same population and treatment. On occasion, there was only a single plant flowering within a population-treatment combination. In these cases, plants were pollinated across population, within treatment, with pollen from the geographically closest population.

Flower, pollen, and nectar samples were collected from live plants. Nectar was collected from a randomly selected subsample consisting of ten plants per population per treatment for a total of up to sixty samples per treatment and up to 240 total samples depending on survivorship and nectar production. Nectar samples were collected using filter paper wicks [43], a method previously used for *C. fasciculata* [37]. This method of utilizing a subsample of ten randomly selected plants per population per treatment was repeated for pollen and flower collection, with a new random subsample selected for each type of sample. Once the plants began to flower, we collected pollen grains by toothpick into microcentrifuge tubes.

### Chemical Analysis

*Chamaecrista fasciculata* accumulation of cadmium was also assessed through examination of the substrate. To determine how much cadmium loss occurred in the substrate due to accumulation, 19–20 pots per treatment containing sand with no plants were added to the second experiment to be utilized as a control to monitor cadmium levels in the sand.

Plants were harvested ninety days after transplantation. We recorded several measures of fitness and growth: at harvest, we recorded flower number and fruit number, final plant height, and after oven-drying all plants at 80°C to constant mass, we collected biomass measurements. These measurements were also collected for plants that died before the set harvest date. Biomass was correlated with other growth measures (Pearson’s correlation coefficients: leaf number (0.57, p = 0.0057), final plant height (0.76, p<0.0001).

After plant harvest, plants were washed with a 10 mM Na_2_EDTA solution (pH 4.1) to remove surface-bound metals, rinsed twice with deionized water, and oven-dried at 80°C to constant mass. After biomass measurements were gathered for each whole plant, material from each plant was divided for elemental analysis: roots, nodules, leaves, stem, mature fruit pods, and mature seeds, along with previously collected flowers, pollen, and nectar, for a total of nine types of sample per plant. The segregated plant material from every plant was measured individually into subsamples of approximately 0.03 g and placed in ceramic crucibles. Samples were placed in a Fisher Scientific Isotemp Programmable Muffle Furnace and ashed at 600°C for 2 hours. Each ashed sample was then placed in a 50 ml Falcon tube, digested in 12.0 ml of 1.0% HNO_3_ solution and centrifuged in an Eppendorf 5702 Centrifuge at 4400 rpm for 5 minutes. After centrifuging, 10 ml of supernatant was removed from each Falcon tube by transfer pipet and placed in a 15 ml Falcon tube. Samples were then analyzed for cadmium concentration using a Thermo Scientific iCE 3300 AA Spectrometer.

For nectar analysis, filter paper wicks were placed in individual 15 ml Falcon tubes and 10.0 ml of 0.1% HNO_3_ solution was poured over each filter paper to reconstitute nectar and separate it from the filter paper. Each sample was vortexed for approximately 30 seconds using a VWR Analog Vortex Mixer and then poured into a 5 ml Whatman Autovial syringeless filter device constructed of polypropylene housing with a 0.45 µm nylon membrane **(**VWR International, #28296-028). Each sample was then filtered through the membrane into a new 15 ml Falcon tube and acidified to 1.0% using Optima grade HNO_3_. Nectar samples were analyzed for cadmium utilizing an Agilent 7500 series ICP-MS. The detection limit of this method was 1 part per billion.

For pollen analysis, samples were collected in 0.6 ml microcentrifuge tubes, and then 0.5 ml of 0.1% HNO_3_ solution was added to each tube. Each sample was sonicated in a Branson 200 Ultrasonic Cleaner for five minutes. Then, 0.4 ml was extracted from each tube by micropipette and poured into a 5 ml Whatman Autovial syringeless filter device constructed of polypropylene housing with a 0.45 µm nylon membrane **(**VWR International, #28296-028), with 3.6 ml of 0.1% HNO_3_ solution added into each filter device to make a 4 ml sample. Each sample was then filtered through the membrane into a new 15 ml Falcon tube and acidified to 1.0% using Optima grade HNO_3_. Pollen was analyzed for cadmium utilizing an Agilent 7500 series ICP-MS.

For elemental analysis of sand, sand from each pot was air-dried and then homogenized after plants were harvested to ensure consistency in the samples. We collected sand samples from pots representing each plant population at each treatment level, as well as sand samples from pots with no plants at each treatment level. The required mass of sand samples varied depending on the amount of cadmium that was added to the sand at the initiation of the experiment, to ensure minimum sample requirements for cadmium detection via AAS. Thus, sand samples from the control treatment were 20.0 mg, samples from 5 mg/kg treatment were 15.0 mg, and samples from the 10 and 15 mg/kg treatments were 5.0 mg. Measured samples were placed into 50 ml Falcon tubes, and a 1.0% HNO_3_ solution was added to each Falcon tube. Samples were sonicated in a Branson 8510 Bath Sonicator for thirty minutes, centrifuged for five minutes in an Eppendorf 5702 Centrifuge at 4400 rpm, and then 10 ml of supernatant was removed from each Falcon tube by transfer pipet and placed in a 15 ml Falcon tube. Samples were analyzed using a Thermo Scientific iCE 3300 AA Spectrometer.

### Statistical Methods

Data were analyzed utilizing **R** 2.11.1 statistical software. Several types of analyses were implemented to assess the response variables of each dataset, including ANOVA, mixed model analyses of variance, and repeated measures ANOVA. For ANOVA, a stepwise method for model fitting was utilized, where models were compared using analysis of deviance to determine the best-fitting model. When implementing mixed models of analysis, a model building framework was employed (package ‘nlme’) [44]. This framework utilized Akaike’s Information Criterion (AIC) to compare and select the best fitting model for each response variable in analysis of germination and growth measures, as well as chemical analyses of plant accumulation. Models included square root transformations of response variables where necessary when normality was violated, and as all models exhibited heterogeneity of variance, a constant variance structure was utilized to model the differing residual variance at each treatment level. The repeated measures ANOVA was also implemented as a mixed-model framework utilizing the model building methods described above, except an autoregressive correlation structure through time was also included in the final model due to violation of independence through repeated measurements on the same individuals.

To assess data on cadmium tolerance and between-population variation in tolerance, mixed model analyses of variance were constructed using model building methods [44]. For all response variables, the full model contained all possible combinations of fixed effects, with block as a random effect, and was compared against simpler models using AIC in order to select the best fitting model. The full models included treatment (Trt), to assess whether the different treatment levels affected each germination, growth, and fitness measure, population (Pop), to assess whether different populations’ germination, growth or fitness measures differed regardless of treatment levels (as assessment of whether inherent variation exists among populations); and a Trt*Pop interaction, to determine whether variation across populations and across treatment levels was exhibited for each of the phenotypic measures. For all models, populations were treated as a fixed effect. For a few response variables we examine the effects of covariates, as follow: time as a covariate for leaf number, biomass as a covariate for flower number, and pollination success rate as a covariate for fruit number.

A tolerance index (TI) was also calculated to measure the ability of plants to grow in a given concentration of cadmium [7], [45], [46]. This index was calculated for the species as a whole at each treatment level, and then for each population at each treatment level following the standard formula:
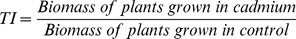



To assess data on cadmium accumulation in roots, nodules, stems, leaves, flowers, fruit pods, seeds, and pollen, mixed model analyses of variance were constructed following model building methods [44], with a separate model constructed for each response variable. As with previous models, the full mixed effects model contained all possible combinations of fixed effects, with block as a random effect, and was compared against simpler models using AIC in order to select the best fitting model. For each response variable, the full model included treatment (Trt), to determine whether the different treatment levels affected the amount of cadmium accumulated in each plant part, population (Pop), to assess whether variation between populations was displayed for cadmium accumulation in each plant part, and a Trt*Pop interaction, to determine whether cadmium accumulation varied among populations at the different treatment levels. A significant Trt*Pop effect would indicate between-population variation in accumulation at the different soil cadmium levels. Due to extremely low survivorship, data from plants grown in the 15 mg/kg treatment could not be included in any statistical analyses including a Trt*Pop term, so a separate, simplified mixed model including only the fixed effect Trt with block as a random effect was run for all response variables to determine whether accumulation across treatments, including the 15 mg/kg treatment, was significantly different. For traits where treatment was found to be significant, we conducted post-hoc tests (Tukey’s Honestly Significant Differences) to compare differences in accumulation across all treatments for each response variable.

We also assessed whether plants partitioned a greater amount of cadmium in their aboveground versus belowground structures. Because some plant parts accumulated less than our detection limit of 1 ppb cadmium, root:shoot concentration ratios could not be accurately calculated. Instead, we averaged the root concentration with nodule concentration for each plant, averaged stem concentration with leaf concentration for each plant, and then obtained the difference between these two values (belowground average concentration – aboveground average concentration = root:shoot concentration difference). A positive value indicated a greater cadmium concentration in the belowground plant structure, whereas a negative value indicated greater accumulation occurred in aboveground plant tissue. A mixed model analysis of variance was then constructed following the methods above, with the root:shoot concentration difference as the response variable. A mixed model analysis of variance including data from the 15 mg/kg treatment, with only the term Trt, was then analyzed for root:shoot concentration difference, followed by Tukey’s Honestly Significant Differences to examine which treatments differed in cadmium accumulation.

To determine whether the soil cadmium concentration differed among pots with plants than those without plants at different treatment levels (likely due to plant uptake), a mixed model of analysis was constructed in the same manner as above, except the data included a seventh “population” comprised of the concentrations of the twenty pots per treatment containing sand with no plants (Ctrl). This same model was also analyzed utilizing a second dataset sorted with all populations of plants grouped together as one population compared to the control pots, to assess whether *C. fasciculata* accumulated a significant amount of cadmium out of the sand across all treatments. This same model was analyzed a third time utilizing a third dataset containing the six plant populations and excluding the control pot population Ctrl and the 15 mg/kg treatment, to determine whether populations of *C. fasciculata* accumulated cadmium differently from each other across treatments. Final model structures for all chemical analyses are listed in (Table S1 **).**

Lastly, we tested whether there was a negative correlation between cadmium accumulation and tolerance, as has been found for some species [47]. Pearson’s correlation coefficients were calculated between biomass (a measure of tolerance and total growth) and shoot cadmium accumulation, as well as biomass and root cadmium accumulation for each population separately [47].

## Results

### Germination, Growth, and Fitness Measures

**Figure 1:**
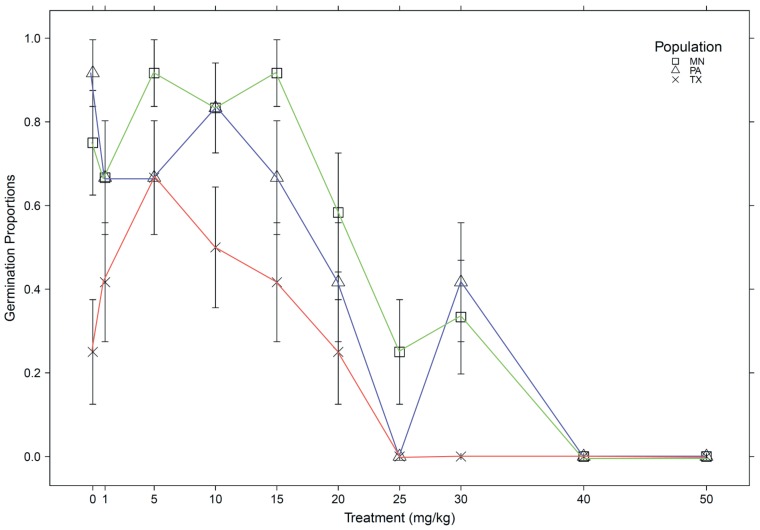
Population and treatment variation in germination. Average germination proportions (with standard errors) between populations MN, PA, and TX across the implemented treatment levels.

Treatment and population significantly affected germination success measured by proportion germinated (*df* = 1, *F* = 65.35, p<0.0001; *df* = 2, *F* = 6.72, p<0.01) and days to germination (Table S1). As a species, *Chamaecrista fasciculata* displayed at least some degree of tolerance in germination to elevated soil cadmium levels. Two of the three populations, TX and MN, exhibited germination stimulation from cadmium by germinating in higher proportions at treatment levels 1 mg/kg–15 mg/kg than in the control treatment (Figure 1). In treatments greater than 10 mg/kg, increasing soil cadmium levels resulted in an overall increase in days to germination. In general, cadmium reduced germination. Between 10–20 mg/kg soil cadmium, there was approximately 50% reduction in germination, depending on the source population. For soil cadmium levels over 30 mg/kg, germination was 100% inhibited. However, multiple populations germinated more quickly on average at 1 mg/kg (MN and PA), 5 mg/kg (MN and PA), and 10 mg/kg (MN and TX) than in the control treatment.

In testing cadmium tolerance throughout the life cycle of the plant, data from plants grown in 15 mg/kg cadmium treatment could not be included in further statistical analyses due to extremely low survival rates compared to the control treatment (*df* = 178, t = 19.41, p<0.0001,), although we include data from survivors in figures for descriptive purposes. Significant treatment and Trt*Pop effects were found to affect survivorship through time (Table S1). Cadmium strongly affected survivorship for the first two weeks of growth in the 5 and 10 mg/kg treatments. The majority of plants (proportion surviving: control: 0.82, 5 mg/kg: 0.62, 10 mg/kg: 0.43, 15 mg/kg: 0.12) that survived this initial three-week period lived through the remainder of the experiment.

Treatment, population, week, Trt*Week, Trt*Pop, Week*Pop, and Trt*Week*Pop significantly affected average weekly leaf number counts (Table S1). Similarly, treatment, population, and Trt*Pop significantly affected average height. After the initial germination stage, elevated soil cadmium levels decreased plant height, biomass and overall change in leaf number over time. There was significant variation between populations for cadmium tolerance between populations as measured by plant height, biomass and leaf number. For example, KY was more robust overall and more tolerant to increases in soil cadmium levels, whereas MN exhibited a less tolerant response. For example, while KS, KY, PA, and MN exhibited a gradual decrease in average biomass as treatment increased, TX and FL displayed a much higher sensitivity to low cadmium levels in their average biomass, with a large decrease in average biomass from control to the 5 mg/kg treatment.

Treatment, population, biomass, and treatment by population were found to significantly affect flower number at harvest (Table S1). Elevated soil cadmium levels decreased flower number in *Chamaecrista fasciculata.* The effects varied between populations, as the KS showed little treatment effect on flowering. Treatment and population significantly affected pollination success rate, although the interaction was not significant (Table S1; Figure S2).

We measured tolerance to cadmium as biomass of cadmium treated plants relative to control plants. Overall, *C. fasciculata* tolerance for cadmium declined as soil concentrations increased (Table 1). Populations had strikingly different tolerance indices, however (Table 1). Populations KS and KY exhibited the greatest tolerance to cadmium at all soil concentrations and the populations from FL and TX exhibited the lowest tolerance.

**Table 1:** Tolerance indices calculated by population and treatment.

Tolerance Indices			
	5 mg/kg	10 mg/kg	15 mg/kg
Overall	0.4678	0.2994	0.1560
FL	0.2344	0.1372	0.0000
KS	0.8438	0.7103	0.4552
KY	0.6712	0.5288	0.3479
MN	0.5389	0.2248	0.0416
PA	0.5171	0.3163	0.1501
TX	0.2752	0.0595	0.0000

### Patterns of Cadmium Accumulation in *Chamaecrista fasciculata*

**Figure 2:**
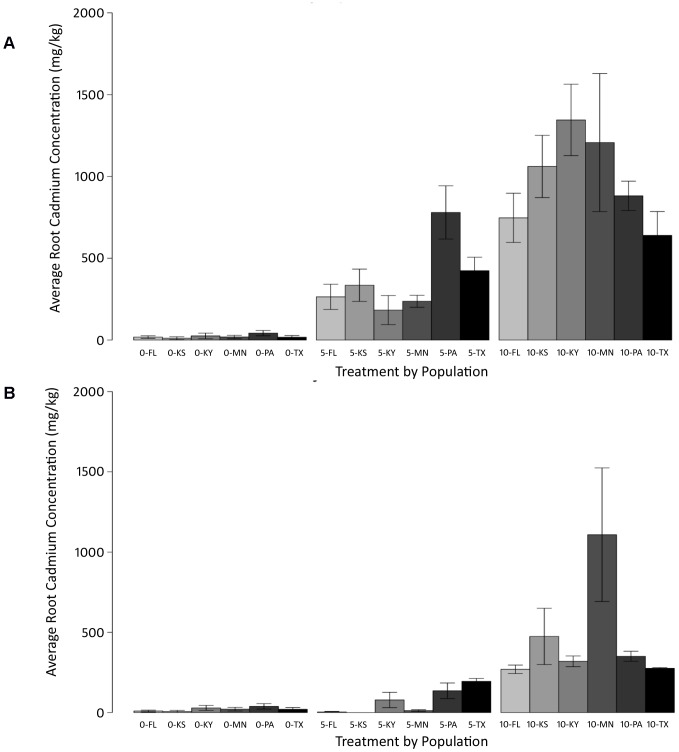
Population variation in cadmium accumulation. A) Average root dry weight cadmium accumulation by each of the six populations of *Chamaecrista fasciculata* in response to elevated soil cadmium levels. B) Average leaf dry weight cadmium accumulation by each of the six populations of *Chamaecrista fasciculata* in response to elevated soil cadmium levels. Treatment levels (0, 5, 10) are in mg/kg.

Because few plants survived in the 15 mg/kg treatment, this treatment was excluded from statistical analyses unless mentioned specifically. Treatment, population, and Trt*Pop significantly affected cadmium concentration in all non-reproductive plant parts of *Chamaecrista fasciculata* (Table S2). Roots accumulated the most amount of cadmium on average in each treatment, followed by nodules, then stems, then leaves (Figure S3). On average, cadmium accumulation ranged from 22–4450 mg/kg dry weight (DW) in roots (Figure 2a), 48–685 mg/kg DW in nodules, 21–2605 mg/kg DW in stems, and 21–2026 mg/kg DW in leaves (Figure 2b), with shoots in all treatment levels almost reaching or exceeding the 100 mg/kg accumulation level conventionally used to define cadmium hyperaccumulation (5 mg/kg treatment: 97.5 mg/kg DW, 10 mg/kg treatment: 578.7 mg/kg DW, 15 mg/kg treatment: 2315.6 mg/kg DW) [22].

As cadmium treatment level increased, cadmium accumulation increased in non-reproductive plant parts including roots, stems and leaves (values in each treatment in Table S3). Nodulation was severely inhibited by elevated soil cadmium levels, with only 3 out of 6 populations producing nodules at the 10 mg/kg treatment level (FL, KS, and KY), and no nodulation at treatment level 15 mg/kg. Statistical analysis of nodule concentrations including Trt*Pop did not include the 10 mg/kg or 15 mg/kg treatments due to overall low numbers of nodulating plants at these treatments. As treatment increased, cadmium accumulation in nodules increased, with average nodule cadmium concentration almost doubling with the increase in treatment from L to M (Table S3; Figure S4). Cadmium accumulation increased significantly in roots, leaves, stems and nodules between the 5 mg/kg and 10 mg/kg treatments, and from the control plants to the 5 mg/kg treatment for all traits except nodules (Table S4). For all plant parts, populations exhibited between-population variation in average cadmium accumulation, as well as between-population variation in cadmium concentration across treatments**.**

**Figure 3:**
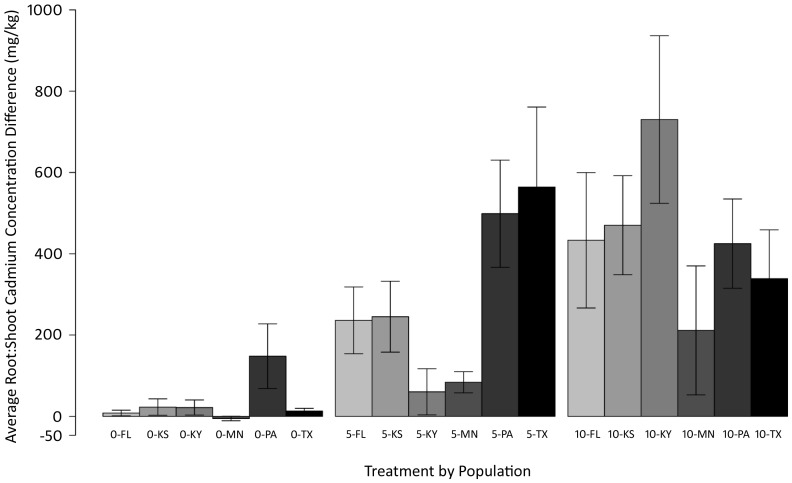
Root and shoot cadmium accumulation. Average difference in root to shoot cadmium accumulation for each of the six populations of *Chamaecrista fasciculata* in response to elevated soil cadmium levels. A positive value indicates that plants in that group accumulated more cadmium in their roots than in the shoots and a negative value indicates the opposite is true. Treatment levels (0, 5, 10) are in mg/kg.

Significant Trt*Pop effects were found for all non-reproductive parts, and significant Trt and Trt*Pop effects were also found for root:shoot concentration difference (Table S2), indicating that populations partitioned their accumulated cadmium differently across treatments and functional organs. Though some individuals accumulated more cadmium into their aboveground structures, on average, plants of *C. fasciculata* accumulated more cadmium into their root structures than shoot structures, with the difference in root:shoot concentration increasing as treatment increased (Figure 3). Between-population variation was found across treatments for difference in root:shoot cadmium concentration.

**Figure 4:**
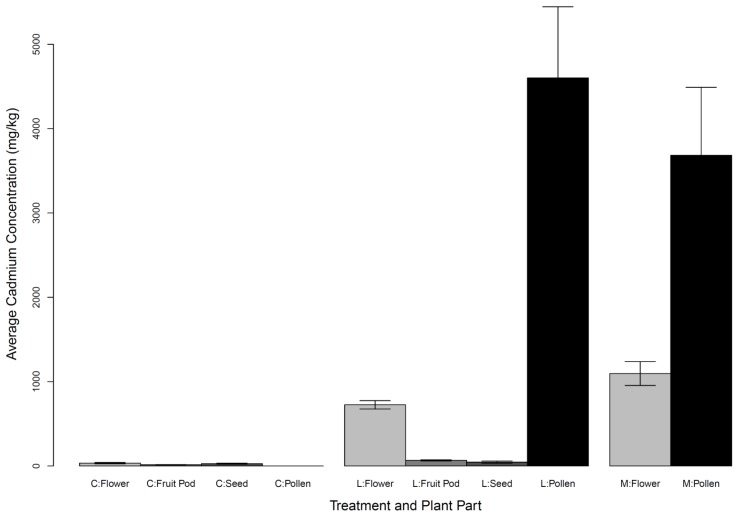
Cadmium accumulation in reproductive structures. Average cadmium accumulation (with standard errors) by flowers, fruit pods, seeds, and pollen of *Chamaecrista fasciculata* across all treatment levels where collected. Measurements are for dry weight for flowers, fruit pods and seeds, and for fresh weight for pollen.Treatment abbreviations: C: 0 mg/kg; L: 5 mg/kg; M: 10 mg/kg.

For reproductive plant parts of *Chamaecrista fasciculata*, treatment had a significant effect on flower and fruit pod cadmium concentration, source population affected flower, seed, and pollen concentration, and Trt*Pop affected flower, fruit pod, and seed concentration (Table S2). Pollen accumulated the highest overall cadmium concentration, followed by flowers, fruit pods, and then seeds (Figure 4). On average, cadmium accumulation ranged from 0–4602 mg/kg fresh weight (FW) in pollen, 34–1096 mg/kg DW in flowers, 14–67 mg/kg DW in fruit pods, and 28–45 mg/kg DW in seeds. Concentrations for pollen and flowers in all treatment levels almost reached or exceeded the 100 mg/kg accumulation level conventionally used to define cadmium hyperaccumulation [22]. No cadmium was detected in nectar samples.

### Cadmium Extraction from Substrate

Treatment and population significantly affected substrate cadmium concentration (Table S2). However, when Tukey contrasts were performed between populations within each treatment, significant differences between populations were not found in any treatment containing cadmium. Substrate cadmium concentration was not found to differ significantly between the control substrate and the substrate with plants (Table S2; Figure S5), indicating that even though plants accumulated high concentrations of cadmium it was not enough to decrease the overall substrate cadmium concentrations at the end of ninety days. The decrease between control substrate and substrate with plants was 0.002 mg/kg for the control treatment, 0.32 mg/kg for the 5 mg/kg treatment, 0.91 mg/kg for the 10 mg/kg treatment, and 3.11 mg/kg for the 15 mg/kg treatment.

### Cadmium Tolerance and Accumulation

To assess the relationship between tolerance and accumulation in *Chamaecrista fasciculata* as a species, Pearson correlations were calculated between biomass (the tolerance measure) and average shoot accumulation (the aboveground accumulation measure) and then calculated between biomass and average root accumulation (belowground accumulation measure). A significant negative correlation was found between biomass and shoot concentration (r = −0.375, p<0.0001). When the relationship between tolerance and accumulation was assessed for each population, no significant correlations were found for populations FL, KS, and KY, and significant negative correlations were found between biomass and shoot concentrations for population PA (r = −0.411, p<0.05), MN (r = −0.664, p<0.0001) and TX (r = −0.741, p<0.0001). A significant negative correlation was found between biomass and root cadmium concentration (r = −0.362, p<0.0001). Correlations were not found to be significant for populations KS and KY, but significant negative correlations were found between biomass and root cadmium concentration for populations FL (r = −0.493, p<0.05), PA (r = −0.394, p<0.05), TX (r = −0.630, p<0.01) and MN (r = −0.685, p<0.0001). The correlations were calculated for the plants across all treatments. We did not find significant correlations between biomass and cadmium accumulation within any of the treatments.

## Discussion

The first step in remediation or restoration involves introduction of propagules to the site, whether as seeds or transplanted seedlings. *Chamaecrista fasciculata* is tolerant to cadmium at this stage, successfully germinating in soil cadmium levels up to 30 mg/kg. Plant growth and fitness provide quantifiable measures of a plant’s continued ability to persist and reproduce in contaminated environments. In *Chamaecrista fasciculata*, some tolerance was exhibited for all growth and fitness measures, although growth and reproduction decreased as soil cadmium levels increased. Plant susceptibility to cadmium toxicity was most significant in the first two weeks of growth in soil cadmium concentrations up to 10 mg/kg, and in the first three weeks of growth in soil cadmium concentrations of 15 mg/kg. After this initial period of establishment, tolerance measured by survivorship was found to be high. Hand-pollination success in *C. fasciculata* decreased on average 15–20% as the soil cadmium levels increased, although pollination was still possible at moderate cadmium levels. Overall plant performance measured by tolerance indices, suggests that plant tolerance decreased by approximately fifteen percent with each increase in treatment. *C. fasciculata* can grow and persist in soil cadmium concentrations up to 10 mg/kg without excessive inhibitions to growth.

In other studies, tolerance indices of plants considered to be acceptable for use in phytoremediation or restoration through revegetation range from approximately 0.24–0.81 in response to elevated cadmium exposure reaching 18 mg/kg in soil and 50 ppm in solution [12], [48], [49]. Tolerance indices of *C. fasciculata* fall within this range when exposed to soil cadmium levels up to 10 mg/kg, with two populations (KS and KY) also falling within this range when exposed to soil cadmium levels up to 15 mg/kg.

Populations within a species may also vary in attributes useful for restoration and remediation. At the germination stage, *Chamaecrista fasciculata* did not exhibit between-population variation for tolerance. However, between-population variation for tolerance was noted in plant growth, as reflected by their tolerance indices (Table 1). For example, populations FL and TX performed relatively poorly in all treatments, whereas populations KS and KY exhibited higher tolerance at all soil cadmium concentrations. Our results suggest that phytoremediation and restoration projects will be more successful if they are preceded by a survey of populations for their tolerance to the contaminant. Populations exhibiting greater tolerance could then be favored during initial restoration.

*C. fasciculata* accumulated high concentrations of cadmium, with shoots in all treatment levels almost reaching or exceeding the 100 mg/kg accumulation level conventionally used to define cadmium hyperaccumulation. Shoot accumulation levels in our work were found to be comparable to the hyperaccumulating species *Thlaspi caerulescens* [22], [50]. Generally, three common traits characterize metal hyperaccumulators: efficient root uptake, efficient root to shoot transport, and a greatly elevated tolerance achieved through internal detoxification [10], [51], [52]. Though *C. fasciculata* exhibited the first two traits, it was not found to exhibit elevated tolerance at higher levels of soil cadmium concentration (>10 mg/kg) and so cannot unreservedly be designated as a hyperaccumulator species.

Between-population variation for accumulating ability and allocation in *Chamaecrista fasciculata* was found for all measured parts of the plant. The populations that accumulated the most cadmium at higher soil concentrations were not the same as those that accumulated the most at lower soil concentrations. Correlations between biomass and root or shoot cadmium concentration were found for some populations, but not others. The two most tolerant populations, KS and KY, exhibited no correlations between biomass and root or shoot cadmium accumulation. This is consistent with previous studies indicating that traits of tolerance and accumulation may be largely independent from each other [10], [53], [54]. While *C. fasciculata* as a species did not exhibit desirable traits of metal sequestration and high cadmium tolerance for use in phytostabilization, one population, FL, accumulated relatively low concentrations of cadmium compared to all other populations at all treatment levels. However, this population exhibited lower tolerance as well. In addition, the KY population accumulated very high levels of cadmium while remaining moderately tolerant in the high cadmium treatment. These characteristics indicate that this population has potential as a cadmium hyperaccumulator. Our findings of between-population variation in accumulation are consistent with previous research that has demonstrated that cadmium accumulation varies widely between populations [55], and that cadmium hyperaccumulation may be a population-specific rather than species-wide trait [25]. To our knowledge, there is no current available information about the distribution or presence of partridge pea populations in cadmium-contaminated soils.

Plants that accumulate heavy metals may expose interacting species to contaminant, resulting in direct toxicity, trophic transfer and disruption of ecosystem services [56]. Excluding nectar, *Chamaecrista fasciculata* accumulated cadmium throughout all plant parts specifically noted for their role in interspecific interactions: stems, leaves, pollen, seeds and root nodules. Pollen, stems, leaves and nectar of *C. fasciculata* are food sources for variety of mammals, birds and insects [37]. Previous research demonstrates that many insect species are unable to detect the presence of metal contamination [56]. Consumption of pollen, stems or leaves of *C. fasciculata* growing in soil cadmium levels of 5 mg/kg or greater could be detrimental to insects or grazing mammals [57], [58]. If *C. fasciculata* will be utilized to phytoremediate an area contaminated by cadmium in levels exceeding 5 mg/kg, efforts should be made to restrict animal access to the site. Our results are also consistent with other studies demonstrating that nodulation is sensitive to increasing heavy metal concentrations [59]. Heavy metal exposure reduces root hair formation and negatively alters root hair morphology, which reduces potential infection sites where nodulation could occur [59].

The finding that less than 1% of nectar samples accumulated cadmium demonstrates that nectar from plants of *C. fasciculata* growing in soil cadmium levels up to 15 mg/kg is safe for consumption by ants, flies and beetles, and poses little risk of trophic transfer or toxicity to insect species. There is evidence that nectar constituents can be adjusted [60], [61], potentially resulting in nectaries that secrete substances to selectively attract desirable organisms to the plant [62], [63], [64]. However, the exact mechanisms of exclusion of heavy metals from nectar are unknown.

As substrate cadmium concentrations did not significantly decrease in sand with plants compared to substrate with no plants, *C. fasciculata* may not extract significant amounts of cadmium from substrate. As a consequence, these plants are poor cadmium extractors but may be good "phytostabilisers", stabilising cadmium in the soil by their roots which accumulate cadmium [65].

### Conclusion

*Chamaecrista fasciculata* exhibited potential as a remediation or restoration species of cadmium-contaminated soils, with germination, growth, and reproductive success exhibited in soils reaching 10 mg/kg. *C. fasciculata* can be expected to encounter similar cadmium soil levels along roadsides, where soil levels can reach approximately 2 mg/kg of bioavailable cadmium; in close proximity to conventional agriculture, (1 to 8 mg/kg cadmium); and near areas where mining and smelting of non-ferrous metals occur, (up to 24 mg/kg) [66]–[68]. As a legume, *C. fasciculata* may be an especially important remediation choice when enhanced soil nitrogen content is important. The potential of *C. fasciculata* for use in remediation or restoration, however, varied significantly across populations, demonstrating the importance of considering seed source when screening populations of *C. fasciculata* for utilization in phytoremediation. Additionally, the development of molecular genetic tools in *C. fasciculata* has led to the recent emergence of this species as a new model genetic legume [41].

## Supporting Information

Figure S1 **Distribution of** ***Chamaecrista fasciculata*** **in the United States.** Seed source states are indicated in red (USDA Plants Database 2009).
(TIF)

Figure S2 **Pollination success across cadmium treatments.** Average pollination success rate (with standard errors) for three populations of *Chamaecrista fasciculata* in response to elevated soil cadmium levels. Treatment abbreviations: C: 0 mg/kg; L: 5 mg/kg; M: 10 mg/kg; H: 15 mg/kg.
(TIF)

Figure S3 **Cadmium accumulation patterns.** Average dry weight cadmium accumulation (with standard errors) by roots, nodules, stems, and leaves of *Chamaecrista fasciculata* across all treatment levels. Treatment abbreviations: C: 0 mg/kg; L: 5 mg/kg; M: 10 mg/kg; H: 15 mg/kg.
(TIF)

Figure S4 **Cadmium accumulation in nodules.** Average nodule cadmium accumulation by each of the six populations of *Chamaecrista fasciculata* in response to elevated soil cadmium levels. Treatment abbreviations: C: 0 mg/kg; L: 5 mg/kg; M: 10 mg/kg.
(TIF)

Figure S5 **Cadmium in substrate.** Comparison of the average soil cadmium concentration at the end of the experiment between substrate with plants and substrate with no plants across all treatment levels. Treatment abbreviations: C: 0 mg/kg; L: 5 mg/kg; M: 10 mg/kg; H: 15 mg/kg.
(TIF)

Table S1 **Final model structures and results for mixed model ANOVAs and repeated measures ANOVA of germination, growth, and fitness measures.**
(DOCX)

Table S2 **Final model results for mixed model ANOVAs of cadmium concentration for each measured plant organ as well as for substrate.**
(DOCX)

Table S3 **Cadmium concentrations (in mg/kg with standard errors) across treatments in different components of** ***Chamaecrista fasciculata.*** No nodules formed in the 15 mg/kg treatment.
(DOCX)

Table S4 **Results for Tukey Contrasts across treatments for chemical analyses of all measured plant parts of** ***C. fasciculata.***
(DOCX)
