# Supplementary figures and images for: Extensive Variation in Cadmium Tolerance and Accumulation among Populations of Chamaecrista fasciculata
Source: PLoS One. 2013 May 7;8(5):e63200. doi: 10.1371/journal.pone.0063200 (PMC3646754; doi:10.1371/journal.pone.0063200)

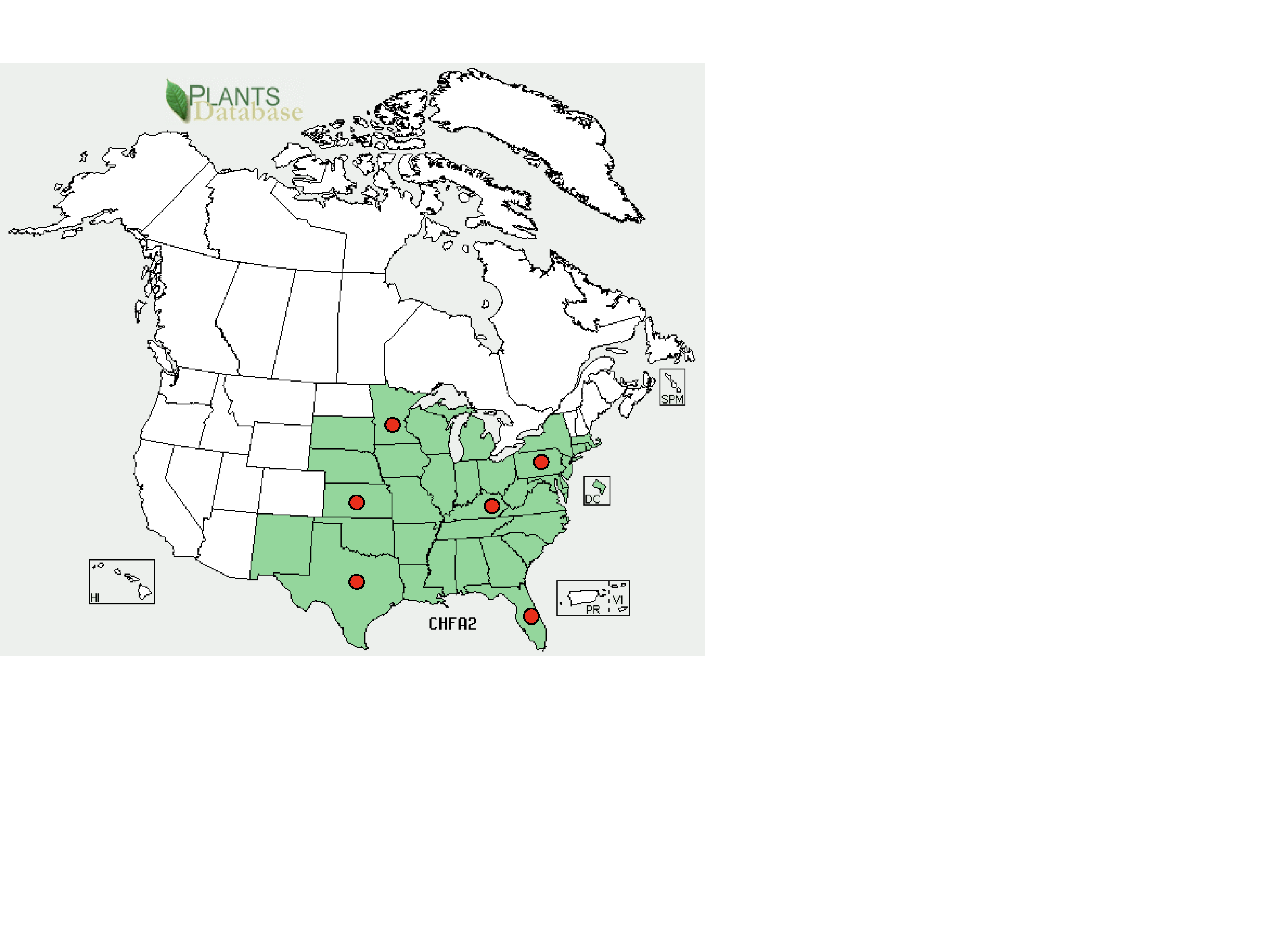

Supplement: Figure S1 — Distribution of Chamaecrista fasciculata in the United States. Seed source states are indicated in red (USDA Plants Database 2009). (TIF) [file pone.0063200.s001.tif]

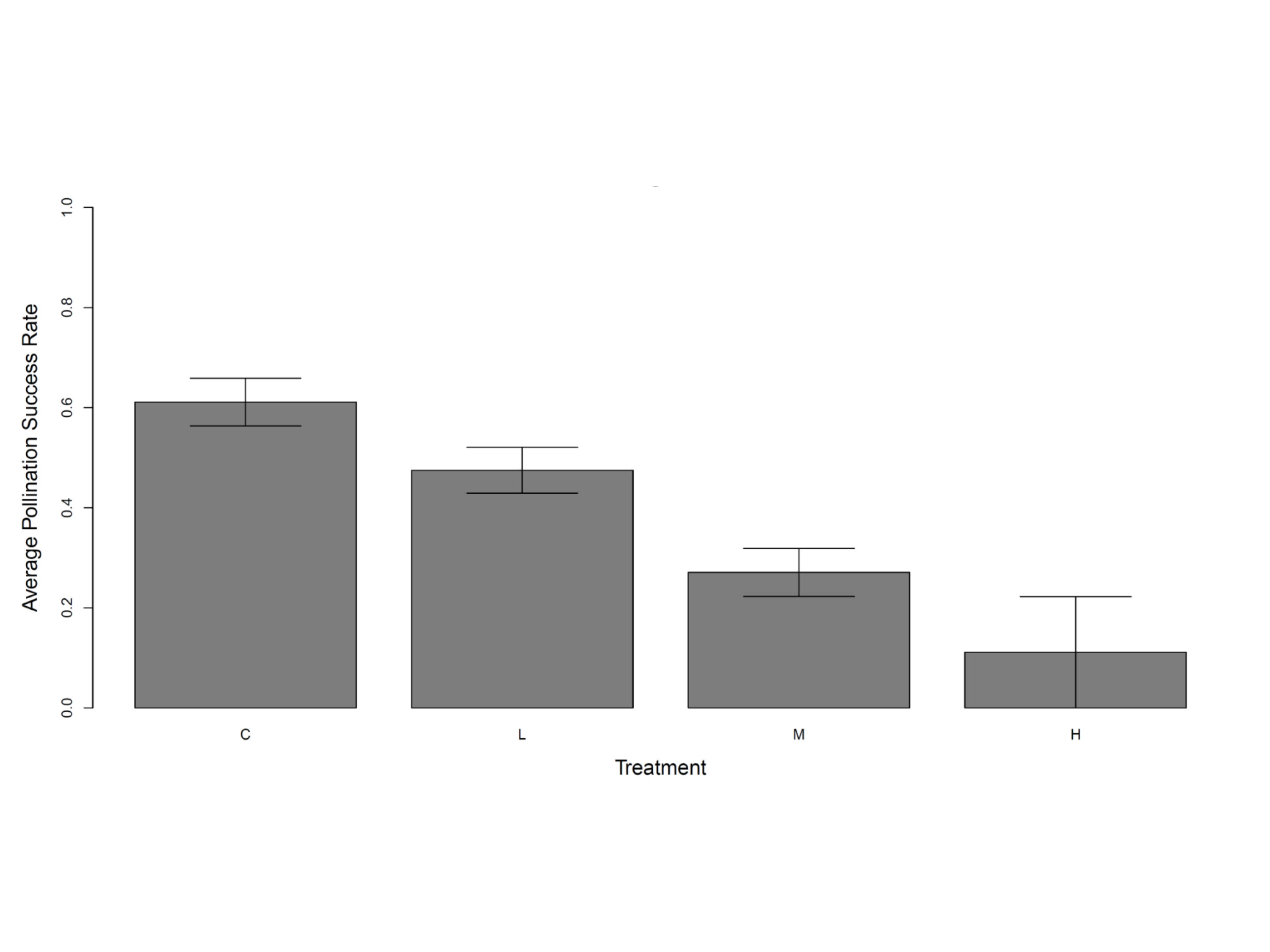

Supplement: Figure S2 — Pollination success across cadmium treatments. Average pollination success rate (with standard errors) for three populations of Chamaecrista fasciculata in response to elevated soil cadmium levels. Treatment abbreviations: C: 0 mg/kg; L: 5 mg/kg; M: 10 mg/kg; H: 15 mg/kg. (TIF) [file pone.0063200.s002.tif]

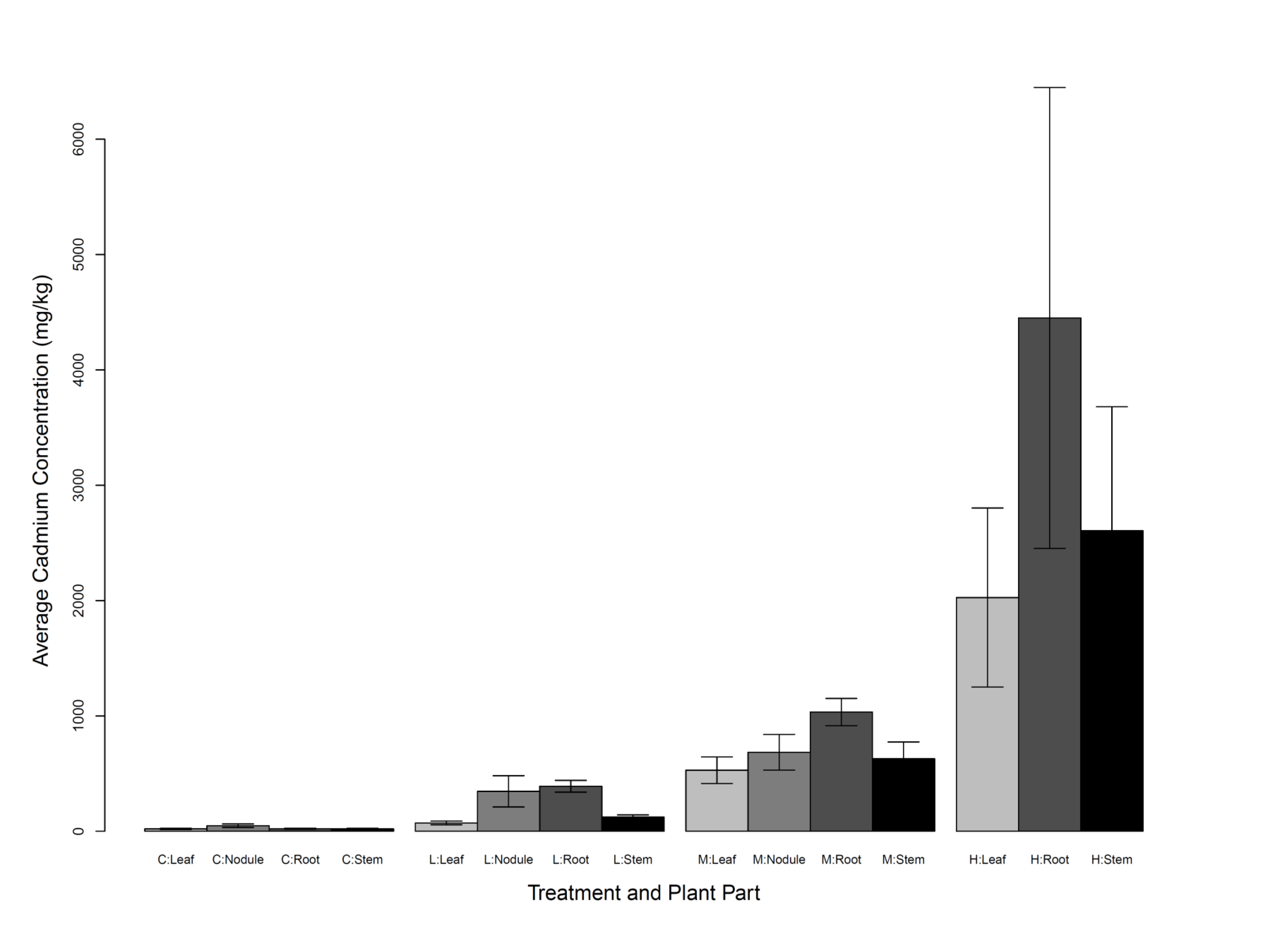

Supplement: Figure S3 — Cadmium accumulation patterns. Average dry weight cadmium accumulation (with standard errors) by roots, nodules, stems, and leaves of Chamaecrista fasciculata across all treatment levels. Treatment abbreviations: C: 0 mg/kg; L: 5 mg/kg; M: 10 mg/kg; H: 15 mg/kg. (TIF) [file pone.0063200.s003.tif]

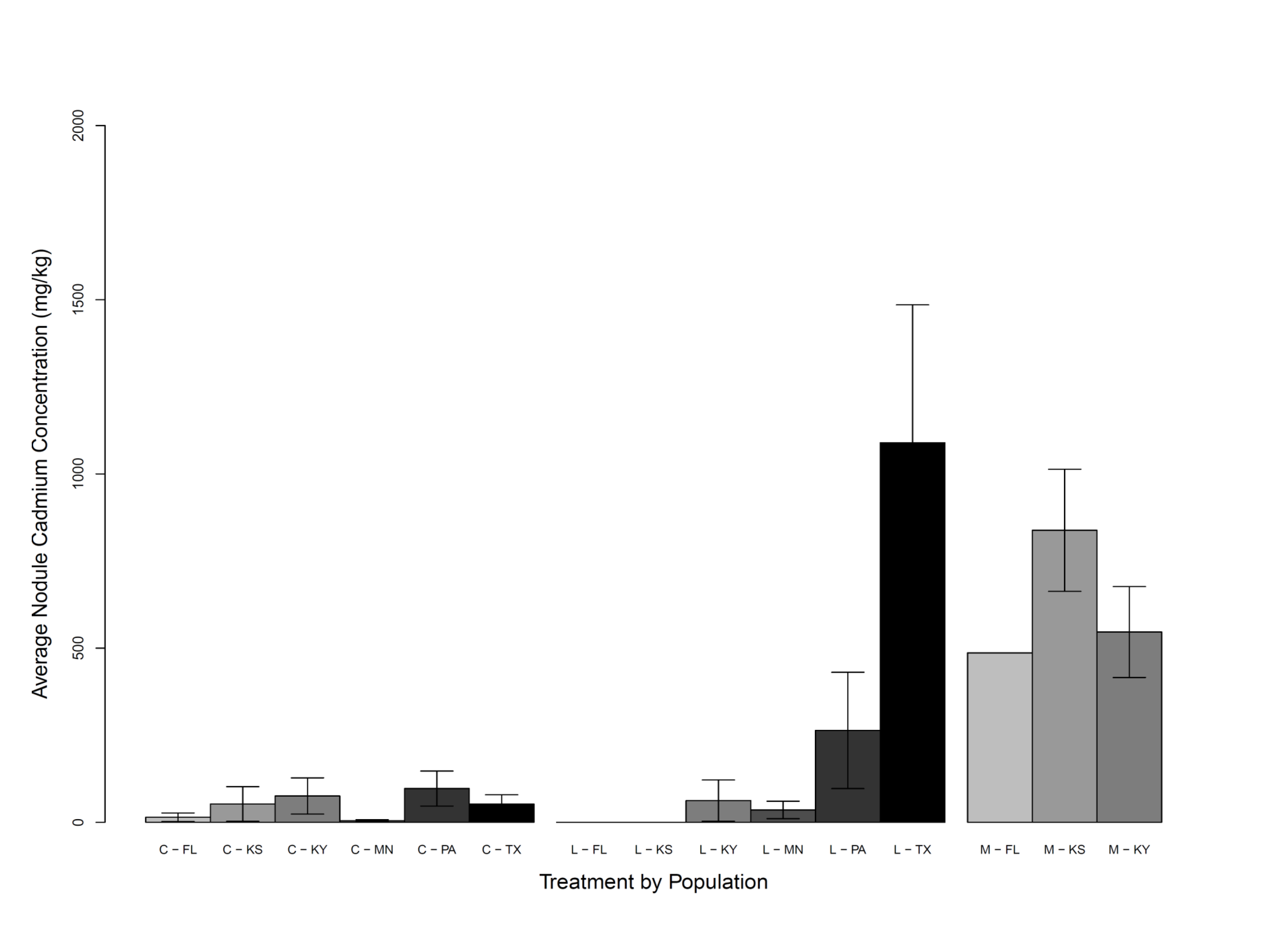

Supplement: Figure S4 — Cadmium accumulation in nodules. Average nodule cadmium accumulation by each of the six populations of Chamaecrista fasciculata in response to elevated soil cadmium levels. Treatment abbreviations: C: 0 mg/kg; L: 5 mg/kg; M: 10 mg/kg. (TIF) [file pone.0063200.s004.tif]

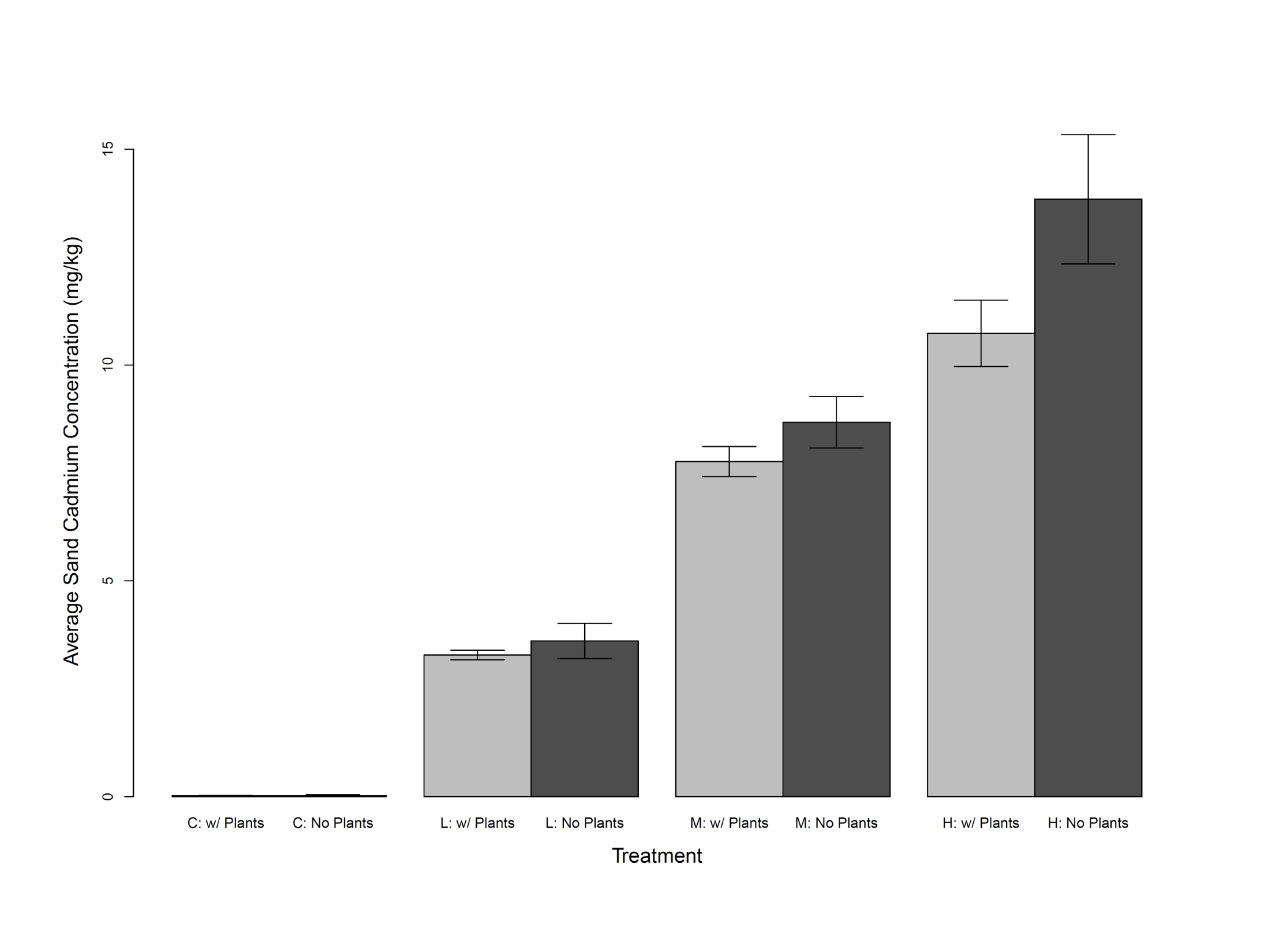

Supplement: Figure S5 — Cadmium in substrate. Comparison of the average soil cadmium concentration at the end of the experiment between substrate with plants and substrate with no plants across all treatment levels. Treatment abbreviations: C: 0 mg/kg; L: 5 mg/kg; M: 10 mg/kg; H: 15 mg/kg. (TIF) [file pone.0063200.s005.tif]
