# Supplementary material for: Extensive Variation in Cadmium Tolerance and Accumulation among Populations of Chamaecrista fasciculata
Source: PLoS One. 2013 May 7;8(5):e63200. doi: 10.1371/journal.pone.0063200 (PMC3646754; doi:10.1371/journal.pone.0063200)
Supplement: Table S1 — Final model structures and results for mixed model ANOVAs and repeated measures ANOVA of germination, growth, and fitness measures. (DOCX) [file pone.0063200.s006.docx]

| Supplemental Table 1: Final model structures and results for mixed model ANOVAs and repeated measures ANOVA of germination, growth, and fitness measures. | | | | | | | | | | | | | | | | |
| --- | --- | --- | --- | --- | --- | --- | --- | --- | --- | --- | --- | --- | --- | --- | --- | --- |
|  | | | | | | | | | | | | | | | | |
| Germination, Growth, and Fitness Measures | Treatment | | Population | | Time | | Biomass | | Pollination Success Rate | | Treatment x Population | | Treatment x Week | | Week x Population | |
|  | df | *F* | df | *F* | df | *F* | df | *F* | df | *F* | df | *F* | df | *F* | df | *F* |
| Days to Germination | 142 | **19.96***** | 142 | **23.29***** | n/a | n/a | n/a | n/a | n/a | n/a | - | - | n/a | n/a | n/a | n/a |
| Leaf Number | 261 | **232.65***** | 261 | **5.71***** | 1710 | **2240.24***** | n/a | n/a | n/a | n/a | 261 | **4.96***** | 1710 | **13.31***** | 1710 | **2.63** |
| Height | 243 | **176.40***** | 243 | **20.15***** | n/a | n/a | n/a | n/a | n/a | n/a | 243 | **7.98***** | n/a | n/a | n/a | n/a |
| Biomass | 210 | **91.41***** | 210 | **40.95***** | n/a | n/a | n/a | n/a | n/a | n/a | 210 | **7.40***** | n/a | n/a | n/a | n/a |
| Flower Number | 209 | **14.40***** | 209 | **8.21***** | n/a | n/a | 209 | **27.55***** | n/a | n/a | 209 | **3.11***** | n/a | n/a | n/a | n/a |
| Pollination Success Rate | 82 | **13.53***** | 82 | **6.31*** | n/a | n/a | n/a | n/a | n/a | n/a | - | - | n/a | n/a | n/a | n/a |
| Fruit Number | 81 | **44.46***** | 81 | **7.90**** | n/a | n/a | n/a | n/a | 81 | **167.53***** | - | - | n/a | n/a | n/a | n/a |
| Days to Death | 453 | **117.72***** | 453 | **37.41***** | n/a | n/a | n/a | n/a | n/a | n/a | 453 | **2.84**** | n/a | n/a | n/a | n/a |
|  |  |  |  |  |  |  |  |  |  |  |  |  |  |  |  |  |
| *****p≤.0001, **p≤.001, *p≤.01, p≤.05** | | |  |  |  |  |  |  |  |  |  |  |  |  |  |  |
| - excluded from final model | |  |  |  |  |  |  |  |  |  |  |  |  |  |  |  |
|  | | | | | | | | | | | | | | | | |
