# Supplementary material for: Extensive Variation in Cadmium Tolerance and Accumulation among Populations of Chamaecrista fasciculata
Source: PLoS One. 2013 May 7;8(5):e63200. doi: 10.1371/journal.pone.0063200 (PMC3646754; doi:10.1371/journal.pone.0063200)
Supplement: Table S2 — Final model results for mixed model ANOVAs of cadmium concentration for each measured plant organ as well as for substrate. (DOCX) [file pone.0063200.s007.docx]

| Supplemental Table 2: Final model results for mixed model ANOVAs of cadmium concentration for each measured plant organ as well as for substrate. | | | | | | |
| --- | --- | --- | --- | --- | --- | --- |
| Cadmium concentration in plant organs: |  |  |  |  |  |  |
|  | Treatment | | Population | | Treatment x Population | |
|  | df | *F* | df | *F* | df | *F* |
| Roots | 120 | **217.52***** | 120 | **2.46** | 120 | **2.18** |
| Nodules | 66 | **9.00*** | 66 | **2.45** | 66 | **4.75**** |
| Stems | 120 | **67.50***** | 120 | **6.14***** | 120 | **2.64*** |
| Leaves | 119 | **48.69***** | 119 | **7.44***** | 119 | **3.95**** |
| Root:Shoot Ratio | 120 | **36.35***** | 120 | n.s. | 120 | **2.09** |
| Flowers | 62 | **494.46***** | 62 | **23.58***** | 62 | **13.07***** |
| Fruits | 30 | **80.68***** | 30 | n.s. | 30 | **5.6*** |
| Seeds | 27 | n.s. | 27 | **3.66** | 27 | **6.76*** |
| Pollen | - | **-** | 38 | **3.75** | - | **-** |
|  |  |  |  |  |  |  |
| Cadmium concentration in substrate |  |  |  |  |  |  |
| Substrate | 146 | **2173.39***** | 146 | **19.46***** | - | - |
| Control substrate vs. Substrate with plants | 163 | **1602.32***** | 163 | n.s. | - | - |
| Substrate with plants | 130 | **1979.79***** | 130 | **29.24***** | - | - |
|  |  |  |  |  |  |  |
| *****p≤.0001, **p≤.001, *p≤.01, p≤.05** |  |  |  |  |  |  |
