# Supplementary material for: Extensive Variation in Cadmium Tolerance and Accumulation among Populations of Chamaecrista fasciculata
Source: PLoS One. 2013 May 7;8(5):e63200. doi: 10.1371/journal.pone.0063200 (PMC3646754; doi:10.1371/journal.pone.0063200)
Supplement: Table S3 — Cadmium concentrations (in mg/kg with standard errors) across treatments in different components of Chamaecrista fasciculata. No nodules formed in the 15 mg/kg treatment. (DOCX) [file pone.0063200.s008.docx]

Supplemental Table 3: Cadmium concentrations (in mg/kg with standard errors) across treatments in different components of *Chamaecrista fasciculata.* No nodules formed in the 15 mg/kg treatment.

|  | Treatment | | | |
| --- | --- | --- | --- | --- |
|  | Control | 5 mg/kg | 10 mg/kg | 15 mg/kg |
| Root | 22.41±4.86 | 390.56±41.04 | 1033.27±117.77 | 4450.07±1998.19 |
| Stem | 20.96±5.06 | 122.93±19.25 | 628.97±145.32 | 2605.14±1075.61 |
| Leaves | 20.63±4.82 | 72.14±15.69 | 529.33± 115.20 | 2026.02±776.04 |
| Nodules | 47.85±15.63 | 346.16±135.64 | 684.77±154.74 | n/a |
