# Supplementary material for: Extensive Variation in Cadmium Tolerance and Accumulation among Populations of Chamaecrista fasciculata
Source: PLoS One. 2013 May 7;8(5):e63200. doi: 10.1371/journal.pone.0063200 (PMC3646754; doi:10.1371/journal.pone.0063200)
Supplement: Table S4 — Results for Tukey Contrasts across treatments for chemical analyses of all measured plant parts of C. fasciculata. (DOCX) [file pone.0063200.s009.docx]

| Supplemental Table 4: Results for Tukey Contrasts across treatments for chemical analyses of all measured plant parts of *C. fasciculata.* | | | | | | |
| --- | --- | --- | --- | --- | --- | --- |
| Tukey Honestly Significant Differences | | | | | | |
|  | 5 mg/kg vs control | 10 mg/ kg vs control | 15 mg/kg vs control | 10 mg/kg vs 5 mg/kg | 15 mg/kg vs 5 mg/kg | 15 mg/kg vs 10 mg/kg |
| Root Concentration | **11.05***** | **18.33***** | **4.05***** | **7.05***** | **2.91*** | n.s. |
| Nodule Concentration | n.s. | **7.91***** | **-** | **3.86***** | **-** | **-** |
| Stem Concentration | **5.45***** | **10.49***** | **4.30***** | **6.71***** | **3.65**** | n.s. |
| Leaf Concentration | **2.64*** | **9.65***** | **4.53***** | **7.51***** | **4.16***** | n.s. |
| Root:Shoot Concentration | **4.64***** | **5.76***** | n.s. | n.s. | n.s. | n.s. |
| Flower Concentration | **11.70***** | **12.45***** | **5.64***** | n.s. | n.s. | n.s. |
| Fruit Pod Concentration | **8.02***** | **-** | **-** | - | **-** | **-** |
| Seed Concentration | n.s. | - | **-** | **-** | **-** | **-** |
|  |  |  |  |  |  |  |
| *****p≤.001, **p≤.01, *p≤.05** |  |  |  |  |  |  |
